# Supplementary material for: Comparison of transformations for single-cell RNA-seq data
Source: Nat Methods. 2023 Apr 10;20(5):665–72. doi: 10.1038/s41592-023-01814-1 (PMC10172138; doi:10.1038/s41592-023-01814-1)
Supplement: Supplementary file 1 — Supplementary Fig. 1, Supplementary Table 1 and mathematical details A1–A3. [file 41592_2023_1814_MOESM1_ESM.pdf]

nature methods

Analysis

https://doi.org/10.1038/s41592-023-01814-1

Comparison of transformations for single-cell RNA-seq data

In the format provided by the authors and unedited

Suppl. Table S1: Overview of the datasets used for the benchmark. The *#Genes* and *#Cells* columns show the number of rows and columns in the count matrix after filtering out rows and columns for which all values were zero. *Perc. Zeros* shows what fraction of all values were 0. *99% Quant* shows the 99% quantile of the counts. *Overdisp.* shows the global overdispersion estimate with *glmGamPoi*.

	#Cells	#Genes	Perc. Zeros	99% Quant	UMI/cell	Overdisp.
**Consistency**						
Hematopoietic Cells	2,838	21,398	87%	12	5,020	0.33
SUM149PT Cells	1,196	25,231	74%	35	54,900	0.14
Lung Epithelium	11,407	20,728	90%	5	7,730	0.17
Pharyngeal Mesoderm	7,581	19,939	79%	19	21,700	0.12
Neural Progenitors	13,572	25,711	87%	7	11,500	0.31
Mouse Mammary	6,969	19,757	89%	6	6,970	0.24
Mouse Aorta	10,477	20,020	86%	8	9,420	0.89
Bovine IVDs	8,231	17,464	90%	6	3,940	1.20
T Helper Cells	10,064	21,153	83%	15	19,300	0.33
T Cells	43,283	23,978	92%	4	5,360	0.53
**Simulation**						
Dyngen	5,000	995	75%	3	291	0.20
Linear Walk	8,569	17,130	90%	5	4,340	2.20
muscat	5,000	999	63%	22	1,830	0.98
Random Walk	8,569	17,192	90%	5	4,820	2.60
scDesign2	2,838	16,199	82%	15	5,170	0.35
**Downsampling (original)**						
mcSCRB	249	16,864	57%	48	59,000	0.47
Fibroblasts	369	16,535	45%	224	199,000	0.82
Fibroblasts 2	737	18,682	48%	181	197,000	0.33
HEK	339	18,746	63%	38	56,100	0.15
siRNA KD	4,298	18,956	56%	106	122,000	0.36
**Downsampling (reduced)**						
mcSCRB	249	16,864	87%	5	5,020	0.32
Fibroblasts	369	16,535	85%	6	5,020	0.19
Fibroblasts 2	737	18,682	88%	5	5,020	0.13
HEK	339	18,746	89%	4	5,140	0.11
siRNA KD	4,298	18,956	88%	5	4,990	0.23

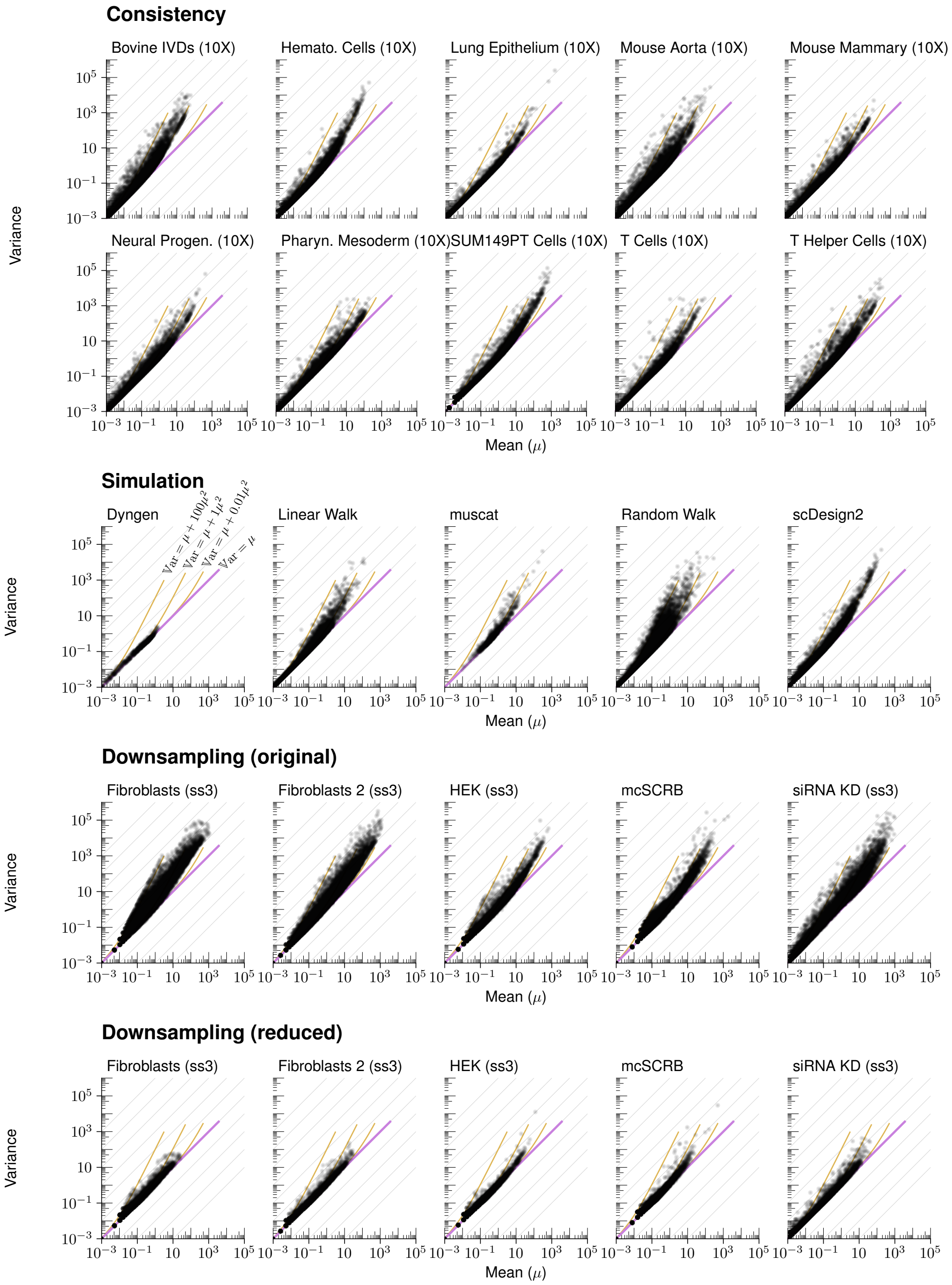

Supplementary Figure S1: Log-log scatter plot of the mean-variance relation across all genes for each dataset. As size factor variations between cells introduce heterogeneity, for each dataset, we filtered out the largest and smallest 25% of cells.

A Mathematical detail

A.1 Variance-stabilizing transformation for a quadratic mean-variance relation

The Gamma-Poisson distribution with mean μ and overdispersion α implies a quadratic mean-variance relation

$$\mathbb{V}\text{ar}[Y] = v(\mu) = \mu + \alpha\mu^2.$$

Our goal is to find a function g for which

$$\mathbb{S}\text{d}[g(Y)] \approx \text{const.}$$

The delta method approximates the standard deviation of a transformed random variable as

$$\mathbb{S}\text{d}[g(Y)] \approx |g'(\mu)|\, \mathbb{S}\text{d}[Y].$$

We can require this to be constant and solve for $|g'(\mu)|$:

$$\begin{aligned} |g'(\mu)|\, \mathbb{S}\text{d}[Y] &= \text{const.} \\ g'(\mu) &= \frac{\text{const.}}{\mathbb{S}\text{d}[Y]} = \frac{\text{const.}}{\sqrt{v(\mu)}} \end{aligned} \tag{1}$$

Given the derivative g', we can use integration to identify the functional form of our transformation (note that without loss of generality, we can ignore the constant, whose value does not affect the variance stabilization property.)

$$\begin{aligned} g(\mu) &= \int \frac{1}{\sqrt{v(\mu)}} \mathrm{d}\mu \\ &= \int \frac{1}{\sqrt{\mu + \alpha\mu^2}} \mathrm{d}\mu \\ &= \frac{2}{\sqrt{\alpha}} \operatorname{asinh}\left(\sqrt{\alpha\mu}\right) \\ &= \frac{1}{\sqrt{\alpha}} \operatorname{acosh}\left(2\alpha\mu + 1\right). \end{aligned} \tag{2}$$

The last two expressions are mathematically equivalent. In the paper, we preferentially use the acosh-based expression since it seems slightly simpler. It is, however, worth noting that in the past, the name asinh transformation has been used (Bartlett, 1947).

If there is no overdispersion ($\alpha = 0$), the acosh transformation reduces to the well-known square root variance stabilizing transformation for Poisson random variables

$$\lim_{\alpha \to 0} g(\mu) = 2\sqrt{\mu}. \tag{3}$$

A.2 Approximating the acosh transformation with the shifted logarithm

The inverse hyperbolic cosine (acosh) transformation from Eq. (1) can also be expressed in terms of the logarithm function,

$$\begin{aligned} g(y) &= \frac{1}{\sqrt{\alpha}} \operatorname{acosh}\left(2\alpha y + 1\right) \\ &= \frac{1}{\sqrt{\alpha}} \log\left(2\alpha y + \sqrt{(2\alpha y + 1)^2 - 1} + 1\right). \end{aligned} \tag{4}$$

We want to approximate this transformation using the shifted logarithm and thus find a, b, and c in

$$h(y) = a + b\log(y + c), \tag{5}$$

so that $h(y) \approx g(y)$.

We aim to find a, b, and c such that for large y, $h(y)$ converges to $g(y)$. We notice that

$$\lim_{y \to \infty} \frac{\sqrt{(2\alpha y + 1)^2 - 1}}{2\alpha y} = 1, \tag{6}$$

and thus for large y

$$\begin{aligned} g(y) &\approx \frac{1}{\sqrt{\alpha}} \log\left(4\alpha y + 1\right) \\ &= \frac{1}{\sqrt{\alpha}} \log\left(y + \frac{1}{4\alpha}\right) + \frac{\log\left(4\alpha\right)}{\sqrt{\alpha}}. \end{aligned} \tag{7}$$

The linear scaling b and the offset a do not influence the variance stabilization; the important insight is that the pseudo-count $y_0 = \frac{1}{4\alpha}$ ensures that the shifted logarithm is most similar to the variance-stabilizing transformation derived using the delta method.

A.3 Delta method-based variance-stabilizing transformation and size factors

Extended Data Fig. S1 demonstrates that delta method-based variance-stabilizing transformations struggle to account for varying size factors.

To incorporate cell-specific size factors in the delta method-based variance stabilizing transformation approach, the counts Y_{ij} are divided by the size factor s_j before applying the transformation: $g(Y_{ij}/s_j)$ (Love et al., 2014). To see the implications of this, it is helpful to look at a decomposition of the variance of a Gamma-Poisson random variable Y:

$$\begin{aligned} Y|Q &\sim \text{Poisson}(Q) \\ Q &\sim \text{Gamma}(\mu, \alpha) \\ Y &\sim \text{Gamma-Poisson}(\mu, \alpha). \end{aligned} \tag{8}$$

In the context of RNA-seq count data, the Poisson level of this hierarchical model represents the technical sampling noise and Q models additional variation. According to the law of total variation

$$\begin{aligned}\mathbb{V}\text{ar}[Y] &= \mathbb{E}[\mathbb{V}\text{ar}(Y|Q)] + \mathbb{V}\text{ar}[\mathbb{E}(Y|Q)] \\ &= \mu + \alpha\mu^2,\end{aligned} \tag{9}$$

where $\mathbb{V}\text{ar}[Y|Q] = \mu$ and $\mathbb{V}\text{ar}[Q] = \alpha\mu^2$.

If we apply the same approach to a model with size factors

$$Y'|Q, s \sim \text{Poisson}(sQ), \tag{10}$$

we find that

$$\begin{aligned}\mathbb{V}\text{ar}[Y'] &= \mathbb{E}[\mathbb{V}\text{ar}(Y'|Q)] + \mathbb{V}\text{ar}[\mathbb{E}(Y'|Q)] \\ &= s\mu' + \alpha s^2\mu'^2 \\ &= \mu + \alpha\mu^2\end{aligned} \tag{11}$$

where $\mu = s\mu'$.

If, however, we want to apply the delta method-based variance-stabilizing transformation to a size factor standardized count

$$X = Y'/s, \tag{12}$$

we find that

$$\begin{aligned}\mathbb{V}\text{ar}[X] &= \frac{1}{s^2}\mathbb{V}\text{ar}[Y'] \\ &= \frac{1}{s^2}(s\mu' + \alpha s^2\mu'^2) \\ &= \frac{1}{s}\mu' + \alpha\mu'^2\end{aligned} \tag{13}$$

The difference between the final line of Eq. (11) and Eq. (13) explains the problem observed when applying the delta method-based variance-stabilizing transformation to correct data where the size factors vary a lot between cells.

References

M. S. Bartlett. The use of transformations. *Biometrics*, 3(1):39, mar 1947. ISSN 0006341X. doi: 10.2307/3001536. URL https://www.jstor.org/stable/3001536?origin=crossref.

Michael I Love, Wolfgang Huber, and Simon Anders. Moderated estimation of fold change and dispersion for RNA-seq data with DESeq2. *Genome Biology*, 15(12):550, 2014. doi: 10.1186/s13059-014-0550-8.
