# Supplementary material for: Comparison of transformations for single-cell RNA-seq data
Source: Nat Methods. 2023 Apr 10;20(5):665–72. doi: 10.1038/s41592-023-01814-1 (PMC10172138; doi:10.1038/s41592-023-01814-1)
Supplement: Supplementary file 2 — Reporting Summary [file 41592_2023_1814_MOESM2_ESM.pdf]

nature portfolio

Corresponding author(s): Wolfgang Huber

Last updated by author(s): Dec 21, 2022

Reporting Summary

Nature Portfolio wishes to improve the reproducibility of the work that we publish. This form provides structure for consistency and transparency in reporting. For further information on Nature Portfolio policies, see our Editorial Policies and the Editorial Policy Checklist.

Statistics

For all statistical analyses, confirm that the following items are present in the figure legend, table legend, main text, or Methods section.

n/a	Confirmed	
☒	☐	The exact sample size (*n*) for each experimental group/condition, given as a discrete number and unit of measurement
☒	☐	A statement on whether measurements were taken from distinct samples or whether the same sample was measured repeatedly
☒	☐	The statistical test(s) used AND whether they are one- or two-sided<br>*Only common tests should be described solely by name; describe more complex techniques in the Methods section.*
☒	☐	A description of all covariates tested
☒	☐	A description of any assumptions or corrections, such as tests of normality and adjustment for multiple comparisons
☐	☒	A full description of the statistical parameters including central tendency (e.g. means) or other basic estimates (e.g. regression coefficient) AND variation (e.g. standard deviation) or associated estimates of uncertainty (e.g. confidence intervals)
☒	☐	For null hypothesis testing, the test statistic (e.g. *F*, *t*, *r*) with confidence intervals, effect sizes, degrees of freedom and *P* value noted<br>*Give P values as exact values whenever suitable.*
☒	☐	For Bayesian analysis, information on the choice of priors and Markov chain Monte Carlo settings
☒	☐	For hierarchical and complex designs, identification of the appropriate level for tests and full reporting of outcomes
☒	☐	Estimates of effect sizes (e.g. Cohen's *d*, Pearson's *r*), indicating how they were calculated

Our web collection on statistics for biologists contains articles on many of the points above.

Software and code

Policy information about availability of computer code

Data collection	No additional software tools were used for data collection.
Data analysis	All code (incl. simulations and data analysis) to reproduce the data analysis is available in the GitHub repository https://github.com/const-ae/transformGamPoi-Paper and stored permanently with Zenodo (https://doi.org/10.5281/zenodo.7504146). The analysis was run using R version 4.1.1. We implemented a package to conduct many of the discussed transformation and made it available on Bioconductor (https://bioconductor.org/packages/transformGamPoi/) in version 3.13.<br><br>We provide an interactive website to explore the benchmark results at https://shiny-portal.embl.de/shinyapps/app/08_single-cell_transformation_benchmark.

For manuscripts utilizing custom algorithms or software that are central to the research but not yet described in published literature, software must be made available to editors and reviewers. We strongly encourage code deposition in a community repository (e.g. GitHub). See the Nature Portfolio guidelines for submitting code & software for further information.

Data

Policy information about availability of data

All manuscripts must include a data availability statement. This statement should provide the following information, where applicable:

- Accession codes, unique identifiers, or web links for publicly available datasets
- A description of any restrictions on data availability
- For clinical datasets or third party data, please ensure that the statement adheres to our policy

Droplet encapsulated RNA	Svensson2017 (CalTech Data Repo entry https://data.caltech.edu/records/1264)
Human hematopoietic cells	Bulaeva 2020 (GEO https://www.ncbi.nlm.nih.gov/geo/query/acc.cgi?acc=GSE130931)
Mouse lung	Angelidis 2019 (GEO https://www.ncbi.nlm.nih.gov/geo/query/acc.cgi?acc=GSE124872)
Human hematopoietic cells	Bulaeva 2020 (GEO https://www.ncbi.nlm.nih.gov/geo/query/acc.cgi?acc=GSE130931)
SUM149PT cell line	No corresponding publication (GEO https://www.ncbi.nlm.nih.gov/geo/query/acc.cgi?acc=GSE142647)
Human lung epithelium	Kathiriya 2022 (GEO https://www.ncbi.nlm.nih.gov/geo/query/acc.cgi?acc=GSE150068)
Mouse pharyngeal mesoderm	Nomaru 2021 (GEO https://www.ncbi.nlm.nih.gov/geo/query/acc.cgi?acc=GSE158941)
Human neural progenitor cells	DeSantis 2021 (GEO https://www.ncbi.nlm.nih.gov/geo/query/acc.cgi?acc=GSE163505)
Mouse mammary	Pal 2021 (GEO https://www.ncbi.nlm.nih.gov/geo/query/acc.cgi?acc=GSE164017)
Mouse aorta	Porritt 2021 (GEO https://www.ncbi.nlm.nih.gov/geo/query/acc.cgi?acc=GSE178765)
Bovine intervertebral discs (IVDs)	Panebianco 2021 (GEO https://www.ncbi.nlm.nih.gov/geo/query/acc.cgi?acc=GSE179714)
Human T helper cells	Qian 2021 (GEO https://www.ncbi.nlm.nih.gov/geo/query/acc.cgi?acc=GSE179831)
Human T cells	Lu 2021 (GEO https://www.ncbi.nlm.nih.gov/geo/query/acc.cgi?acc=GSE184806)
Human pancreas	Baron 2016 (BioC package https://doi.org/doi:10.18129/B9.bioc.scRNAseq)
JM8 cells	Bagnoli 2018 (GEO https://www.ncbi.nlm.nih.gov/geo/query/acc.cgi?acc=GSE103568)
HEK cells	Hagemann 2020 (ArrayExpress https://www.ebi.ac.uk/arrayexpress/experiments/E-MTAB-8735/)
Fibroblasts (1)	Hagemann 2020 (ArrayExpress https://www.ebi.ac.uk/arrayexpress/experiments/E-MTAB-8735/)
Fibroblasts (2)	Larsson 2021 (ArrayExpress https://www.ebi.ac.uk/arrayexpress/experiments/E-MTAB-10148/)
siRNA Knockdown (KD)	Johnsson 2022 (Github https://github.com/sandberg-lab/lncRNAs_bursting/tree/main/data)

Human research participants

Policy information about studies involving human research participants and Sex and Gender in Research.

Reporting on sex and gender	Not applicable
Population characteristics	Not applicable
Recruitment	Not applicable
Ethics oversight	Not applicable

Note that full information on the approval of the study protocol must also be provided in the manuscript.

Field-specific reporting

Please select the one below that is the best fit for your research. If you are not sure, read the appropriate sections before making your selection.

☒ Life sciences ☐ Behavioural & social sciences ☐ Ecological, evolutionary & environmental sciences

For a reference copy of the document with all sections, see nature.com/documents/nr-reporting-summary-flat.pdf

Life sciences study design

All studies must disclose on these points even when the disclosure is negative.

Sample size	No sample size calculation was performed as we reanalyzed publicly available datasets and the chosen datasets consisted of hundreds of cells. The number of datasets for the downsampling and simulation benchmark was limited by the availability of appropriate tools / datasets. For the consistency benchmark, a small pilot benchmark confirmed that 10 datasets were sufficient to differentiate the performance of the transformations.
Data exclusions	No specific data was excluded. We applied basic quality control thresholds to the single-cell data to filter out dead cells and droplets that did not contain cells, as reported in the reproducible code.
Replication	All results can be reproduced using the code in https://github.com/const-ae/transformGamPoi-Paper
Randomization	Randomization was not relevant for our study because the computational nature of our study means that we did not perform an intervention

Randomization	so randomization is per definition not applicable.
Blinding	Blinding was not relevant for our study because the computational nature of our study means that we did not perform an intervention so blinding is per definition not applicable.

Reporting for specific materials, systems and methods

We require information from authors about some types of materials, experimental systems and methods used in many studies. Here, indicate whether each material, system or method listed is relevant to your study. If you are not sure if a list item applies to your research, read the appropriate section before selecting a response.

Materials & experimental systems

n/a	Involved in the study
☒	☐ Antibodies
☒	☐ Eukaryotic cell lines
☒	☐ Palaeontology and archaeology
☒	☐ Animals and other organisms
☒	☐ Clinical data
☒	☐ Dual use research of concern

Methods

n/a	Involved in the study
☒	☐ ChIP-seq
☒	☐ Flow cytometry
☒	☐ MRI-based neuroimaging
